# Ferroptosis and epilepsy: bidirectional pathogenic links and therapeutic implications

**DOI:** 10.3389/fneur.2025.1635441

**Published:** 2025-07-30

**Authors:** Jing Xu, Xinning Dong, Mu Yuan, Xin Chen, Haifeng Shu, Sixun Yu

**Affiliations:** ^1^Department of Neurosurgery, Western Theater General Hospital, Chengdu, China; ^2^College of Medicine, Southwest Jiaotong University, Chengdu, China; ^3^Department of Hyperbaric Oxygen Medicine, Western Theater General Hospital, Chengdu, China

**Keywords:** ferroptosis, epilepsy, iron metabolism, lipid peroxidation, reactive oxygen species

## Abstract

Ferroptosis is a distinctive form of regulated cell death that is closely associated with various neurodegenerative disorders. In recent years, an increasing number of studies have demonstrated the crucial role of ferroptosis in the development and progression of epilepsy. Firstly, this article will review the existing research on the specific biological mechanism of ferroptosis in nerve injury, particularly in epilepsy, encompassing iron metabolism disorders and alterations in the expression of ferroptosis-related proteins. Secondly, with regards to treatment, this article will explore the application of ferroptosis inhibitors in antiepileptic therapy and their potential therapeutic effects. Additionally, it will focus on investigating the interaction between ferroptosis and existing antiepileptic drugs as well as the potential impact of strategies regulating ferroptosis on epilepsy treatment. Finally, we will evaluate both the progress made and limitations encountered in current research while proposing possible future directions for further exploration at the intersection of ferroptosis and epilepsy fields. These studies not only contribute to a better understanding of epileptic pathological mechanisms but also hold promise for providing novel insights and strategies for treating epilepsy.

## 1 Introduction

Epilepsy is a chronic neurological disorder characterized by recurrent seizures, which arise from aberrant and generalized electrical activity in the brain (1). Globally, epilepsy affects approximately 50 million individuals and has a prevalence of 0.5 to 1% among children, significantly impairing their quality of life. Epilepsy remains insufficiently understood by many, with certain social contexts stigmatizing it as “abnormal” or “defective.” Moreover, apart from the individual level impact, the resource-intensive nature of epilepsy treatment and necessary support services during rehabilitation substantially contribute to the social and economic burden (2). The etiology of epilepsy is intricate and diverse, encompassing genetic factors, brain injury, infection, and structural abnormalities of the central nervous system. However, despite significant advancements in the diagnosis and treatment of epilepsy, approximately 30–40% of patients remain unresponsive to existing antiepileptic drugs (3). Consequently, there exists an urgent imperative to explore novel therapeutic targets and strategies.

Ferroptosis, characterized as an iron-dependent form of programmed cell death, represents a regulated mechanism of cellular demise triggered by iron-catalyzed lethal lipid damage. It significantly diverges from conventional pathways of cell death (such as apoptosis and autophagy) and was initially reported and elucidated by Dixon et al. (4, 5). The regulation of ferroptosis signaling pathways encompasses the control of iron homeostasis, RAS pathway, and cystine transport pathway. Dysregulation in these pathways can contribute to the occurrence of ferroptosis. Although the underlying mechanism remains elusive, ferroptosis exhibits several distinctive features: ① Alterations in cell morphology are prominent during ferroptosis, characterized by reduced mitochondrial size, increased membrane density, diminished or absent mitochondrial cristae, and potential disruption of the outer membrane. However, discernible changes in nuclear morphology are not observed (6). ② Metabolic perturbations: Enhanced lipid peroxidation leading to excessive reactive oxygen species (ROS) accumulation represents a hallmark feature of ferroptosis. Concurrently, intracellular levels of ferric ions also accumulate (7). ③ Impaired glutathione peroxidase function: Deficiency in the membrane lipid repair enzyme glutathione peroxidase (GPX4) results in ROS buildup on membrane lipids (8). Although the specific regulatory networks governing these features remain elusive, ferroptosis primarily encompasses three factors: aberrant iron ion metabolism, depletion of REDOX glutathione (GSH)/ GPX4/ cystine-glutamate transporter system (System Xc^−^), and dysregulated lipid peroxidation (9, 10). In recent years, the involvement of ferroptosis in the pathogenesis of various neurological disorders has garnered significant attention, encompassing traumatic brain injury, stroke, Alzheimer’s disease, Parkinson’s disease, Huntington’s disease, and brain tumors (11–16). Ferroptosis has also been implicated in epilepsy (17); however, its precise role and underlying mechanisms in epileptogenesis remain elusive. Elucidating the regulatory mechanisms governing iron-induced cell death in epilepsy could offer novel avenues for prevention and treatment strategies. This review article aims to comprehensively explore the mechanistic aspects of ferroptosis and its implications in epilepsy to provide valuable insights into potential therapeutic targets.

## 2 Biochemical features of ferroptosis

Ferroptosis is characterized by three core pathological processes: iron dysregulation, glutathione depletion, and lipid peroxidation.

### 2.1 Iron metabolism dyshomeostasis

**Figure 1:**
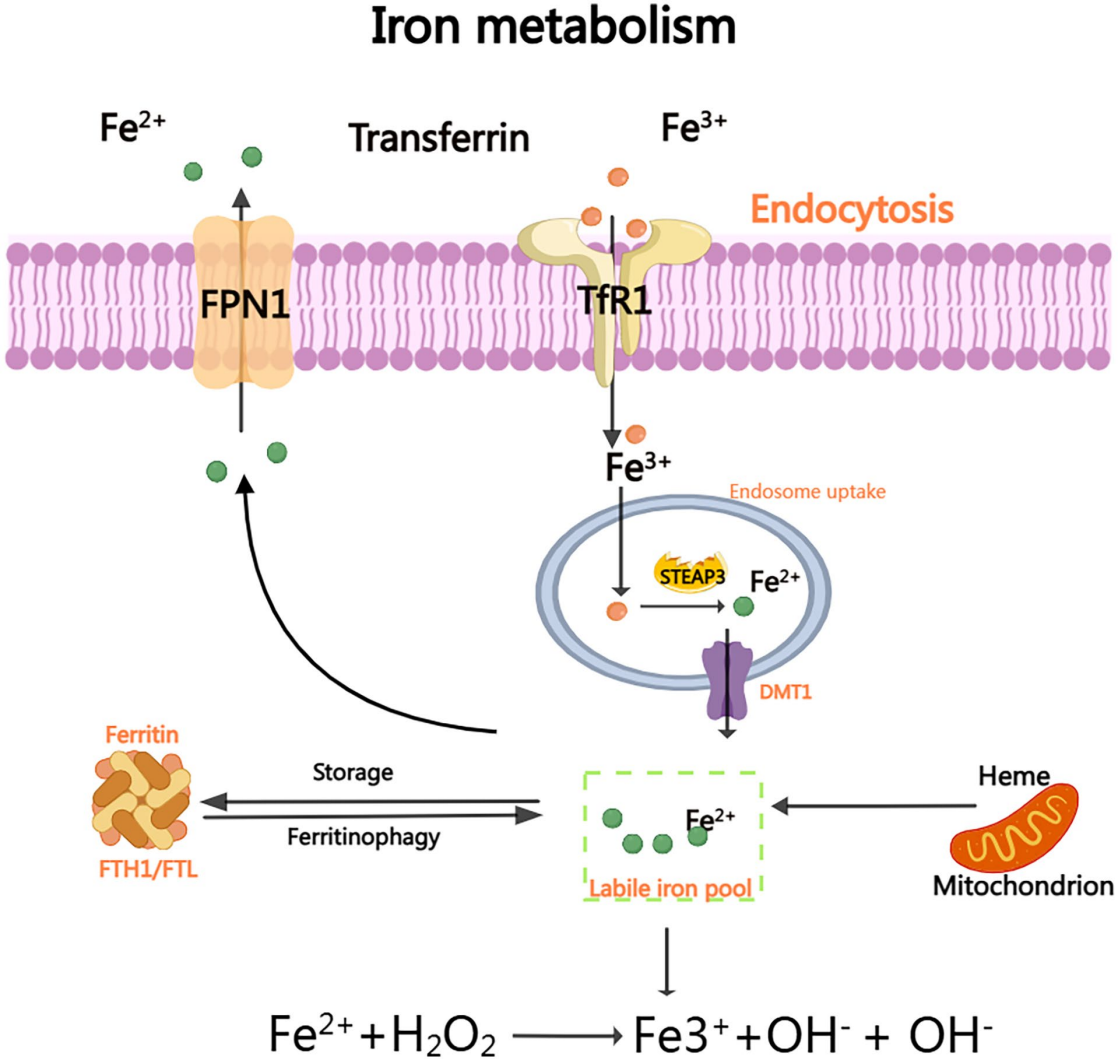
Iron metabolism: The efficient delivery of Fe^3+^ to various organs is facilitated by the binding of plasma transporter TF. Upon binding to TFR1, TF facilitates the smooth transfer of iron ions into the cell interior and subsequent release of Fe^3+^. Subsequently, STEAP3 reduces Fe^3+^ and converts it to accessible Fe^2+^ within the cytoplasm. Through the DMT1 channel, Fe^2+^ can enter the cytoplasm and form a stable iron pool (LIP). Excess unbound Fe^3+^ can be exported outside the cell via FPN and subsequently oxidized back to Fe^3+^. As a crucial substrate for generating hydroxyl radical (OH) and hydroxide ion (OH-) through Fenton reaction, Fe^2+^ plays a pivotal role.

Cellular iron homeostasis is maintained through transferrin (Tf)-mediated transport. Fe^3+^ binds to Tf forming holo-Tf, which undergoes endocytosis via TfR1 on brain microvascular endothelial cells (BMECs). After reduction to Fe^2+^ by STEAP3/DCYTB, iron enters the cytoplasm via DMT1, forming the labile iron pool (LIP). Excess Fe^2+^ catalyzes hydroxyl radical (•OH) generation through Fenton reactions, driving peroxidation of polyunsaturated fatty acids (PUFAs). Iron chelators (e.g., deferoxamine) and suppression of iron-regulatory genes (IREB2, TFRC) inhibit ferroptosis by reducing iron availability (18–25). The signaling mechanism is illustrated in Figure 1.

### 2.2 System xc^−^-GPX4 axis failure and lipid peroxidation

**Figure 2:**
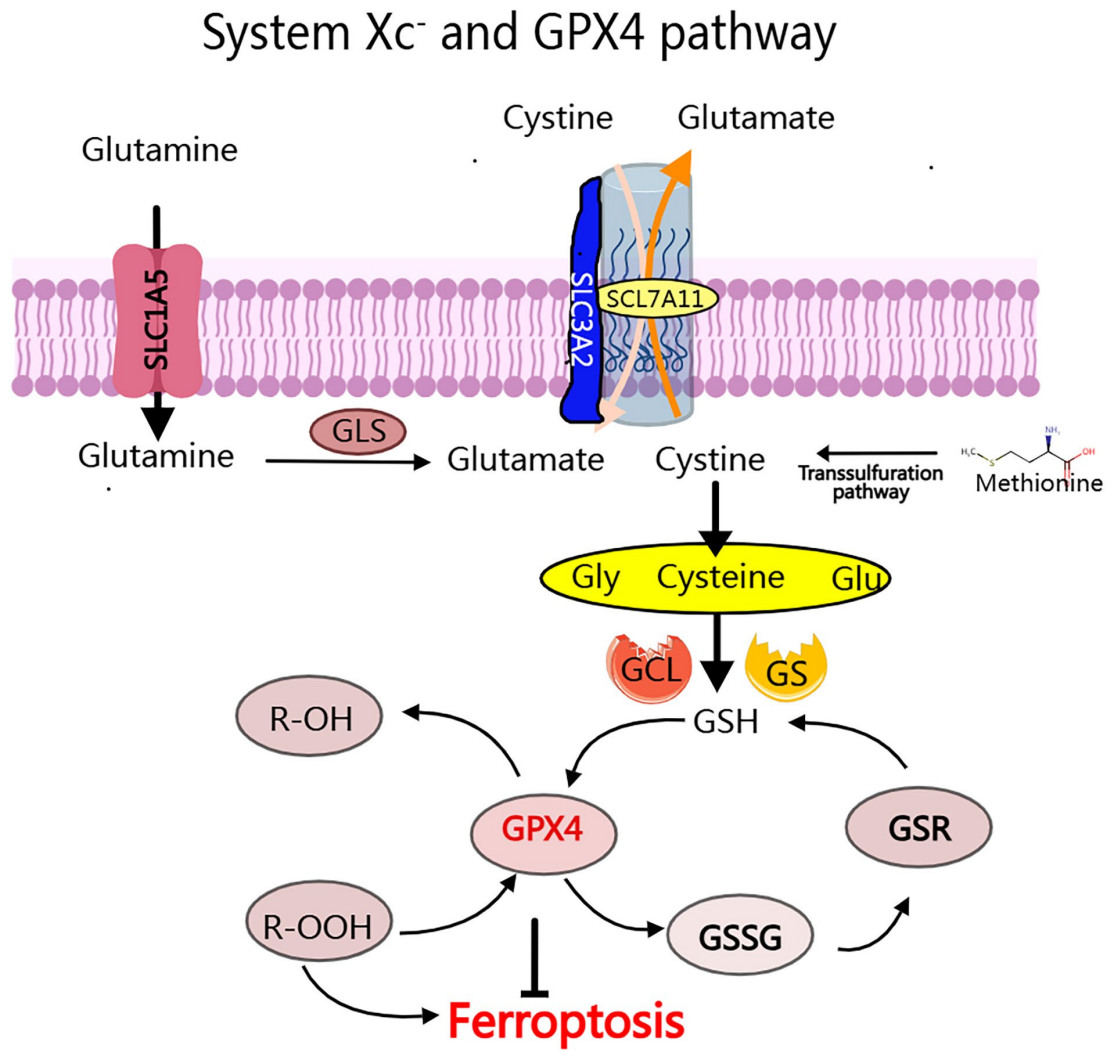
The System Xc- and GPX4 pathway: The conversion of cytoplasmic cysteine to glutathione (GSH) for tripeptide synthesis primarily occurs through the process ocess of glutamate-cysteine exchange, which involves a two-step enzymatic reaction: (1) Glutamic acid cysteine transaminase (GCL), also known as y-glutamyl cysteine synthetase (y-GCS); (2) Glutathione synthetase (GS). The major regulators of iron ptosis include GPX4, GSH, and System Xc-.

**Figure 3:**
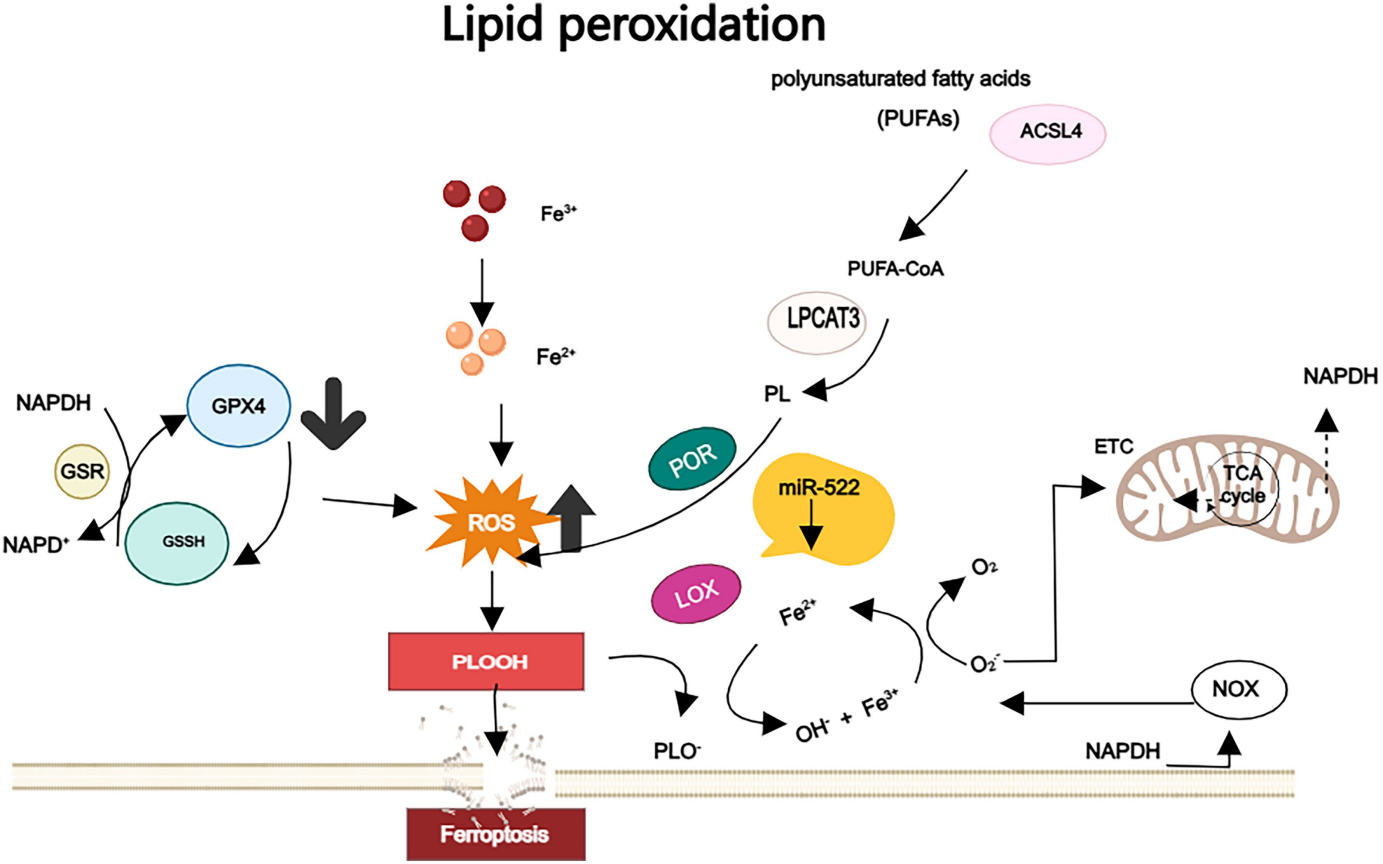
The lipid peroxidation: PUFAs are esterified to membrane phospholipids and subsequently react with ROS, thereby promoting cell ferroptosis. GPX4 uses GSH as a cofactor to enzymically reduce the lipid peroxides of polyunsaturated fatty acids to non-toxic lipid alcohols. Loss or inactivation of GPX4 results in the accumulation of lipid peroxides above normal levels, and in the presence of Fe^2+^ these lipid peroxides generate highly oxidized alkoxy groups. These alkoxy groups have the ability to directly damage adjacent PUFAs through free radical-mediated chain reactions, resulting in severe membrane damage.

The System Xc^−^-GPX4 axis plays a crucial role in maintaining cellular redox homeostasis (26). System Xc^−^ is responsible for importing cystine, which is subsequently reduced to cysteine, facilitating the synthesis of GSH. This GSH serves as a critical cofactor for GPX4, an enzyme that converts toxic lipid hydroperoxides, such as polyunsaturated fatty acid hydroperoxides (PUFA-OOH), into benign alcohols. When GPX4 is inhibited (for example, by RSL3) or when System Xc^−^ becomes dysfunctional, GSH levels are depleted (27, 28) (Figure 2). This depletion leads to the accumulation of lipid ROS through Fe^2+^-catalyzed peroxidation reactions (29). Additionally, it causes mitochondrial damage, characterized by the loss of cristae and membrane rupture, ultimately resulting in the collapse of membrane integrity through alkoxyl radical chain reactions (30). Notably, PUFAs in neuronal membranes are primary targets for lipid peroxidation, with ROS derived from mitochondrial metabolism and NADPH oxidases further amplifying oxidative stress (28) (Figure 3).

### 2.3 Mevalonate pathway: a metabolic gatekeeper of ferroptosis

The mevalonate (MVA) pathway—best known for cholesterol synthesis—is also a pivotal brake on ferroptosis. HMG-CoA reductase generates isopentenyl pyrophosphate that fuels two anti-ferroptotic arms: ① synthesis of coenzyme Q10, whose reduced form, recycled by FSP1 at the plasma membrane, scavenges lipid-peroxyl radicals independently of GPX4; inhibition of HMG-CoA reductase depletes CoQ10, weakens FSP1 activity and markedly increases ferroptotic sensitivity (31, 32); ② isopentenylation of tRNA^Sec, a modification required for efficient GPX4 translation, so limiting MVA flux lowers GPX4 and elevates lipid-ROS (33). In addition, geranylgeranyl pyrophosphate prenylates small GTPases that regulate iron uptake and phospholipid remodeling; loss of prenylation disrupts iron homeostasis and further primes cells for ferroptosis (34). Hence, intact MVA metabolism sustains both the FSP1-CoQ10 shield and GPX4 abundance, acting as a central metabolic checkpoint that dictates ferroptosis sensitivity.

## 3 Association between ferroptosis and epilepsy

Epilepsy is a chronic, recurrent disorder of brief transient dysfunction of brain function, which seriously affects the quality of life of patients and has the risk of causing unexpected death and sudden death (35, 36). Since current anti-epileptic drugs are mainly used for the control of seizures, there is still a great urgency for basic pathophysiological research (37, 38). Studies have shown that epileptiform seizures may initially originate from ion channel dysfunction caused by various stress events such as central nervous system injury, and that astrocyte proliferation and inflammatory responses play important roles in the pathological process of neuronal network excitation and inhibition imbalance, which is a self-perpetuating process. Regularly occurring excessive synchronized abnormal discharges of cortical neurons indicate the formation of epilepsy. Repetitive abnormal neuronal discharges can further lead to various pathological changes at the cellular level, including excessive oxidation. This process forms a “hypoxia, oxidative stress and inflammation” triad, which is a vicious cycle of neuronal activation, leading to systemic brain dysfunction (39, 40).

### 3.1 Potential involvement of ferroptosis mechanisms in the pathogenesis of epilepsy

Iron is an indispensable trace element for human growth and development, playing crucial roles in various REDOX reactions such as oxygen transportation, cellular oxidative respiratory chain, tricarboxylic acid cycle, and DNA biosynthesis (41, 42). Additionally, iron closely associates with myelin formation and catecholamine neurotransmitter metabolism in the nervous system (43, 44). Henceforth, meticulous regulation of iron metabolism is imperative within the human body. The most prevalent neurological disorders associated with abnormal iron metabolism are hemorrhagic stroke and post-traumatic epilepsy (PTE) (45, 46), which also exhibit the highest incidence of secondary epilepsy among neurological diseases. Ferroptosis, a newly recognized form of regulated cell death characterized by excessive lipid peroxidation and ROS production due to iron overload, has been observed in various neurological disorders including epilepsy (47). Therefore, based on chronological extrapolation, ferroptosis can be considered a significant pathological link leading to iron overload-induced epilepsy.

Iron overload is frequently implicated when conducting a comprehensive patient history of epilepsy and examining the impact of stressful brain events, such as PTE, which is characterized by vascular extravasation of red blood cells and elevated hemoglobin levels within the central nervous system. The breakdown of hemoglobin releases a substantial quantity of iron ions, leading to significant reduction in cell viability, superoxide dismutase and glutathione levels, increased generation of reactive oxygen species, lipid peroxidation and malondialdehyde levels, as well as upregulation in the expression of ferroptosis-related proteins that induce mitochondrial ultrastructural changes. Ultimately, this cascade culminates in the exacerbation of ferroptosis process (48–50).

Animal studies suggest that iron overload may underlie the pathophysiology of epilepsy and correlate with seizure onset and severity. For instance, cortical injection of hemoglobin or iron salts (FeCl_3_) into rats can induce chronic seizures, effectively replicating the characteristics of PTE in humans (51). On one hand, iron accumulation can upregulate the expression of Nav1.1 and Nav1.6 in the cortex and hippocampus, thereby modulating neuronal excitability (52, 53). On the other hand, excessive iron levels within cells after FeCl_3_ injection in the rat cerebral cortex can aberrantly activate the mitochondrial oxidative phosphorylation pathway. Excess Fe^2+^ can donate electrons to H_2_O_2_ and O_2_, resulting in substantial production of O_2_ − and OH free radicals which may cause lipid peroxidation of neuronal membranes as well as accelerated generation of guanidine compounds in the brain, ultimately leading to epileptic conditions (51). Injection of nanoscale iron into the cerebral cortex can induce chronic epilepsy in mice and replicate brain damage caused by microbleeds, resulting in varying degrees of spontaneous epileptiform events (51). The severity of epileptiform events is correlated with the reduction of gamma-aminobutyric acid (GABA) neurons and impairment of cerebral blood flow autoregulation in the hemisphere injected with iron (54). In the human study, researchers analyzed transferrin saturation in 130 patients with epilepsy compared to 128 sex- and age-matched control subjects without epilepsy to investigate whether iron overload is a predisposing factor for epilepsy. The results revealed that the mean transferrin saturation of the epilepsy group was significantly higher than that of the control group (55). Zimmer’s study also identified overexpression of iron regulatory genes in TSC patients, FCD IIb patients, and Tsc1GFAP − / − mice, suggesting that early and persistent activation of antioxidant signaling and disruption in iron metabolism are pathological markers for FCDIIb and TSC (56). A retrospective study found significantly elevated levels of transferrin in epileptic children with a history of encephalitis compared to normal controls, indicating that ferroptosis plays a crucial role in oxidative stress-induced epileptiform activity during infantile brain inflammation due to high oxygen consumption, low antioxidant capacity, increased brain iron content, and abundant PUFAs in neuronal membranes (55). Kobrinsky et al. (57) through a case–control study, demonstrated that anemia can raise the threshold for first febrile seizures (FS), while iron deficiency may prevent their onset.

The MVA pathway provides a basis for exploring the potential involvement of ferroptosis in the development of epilepsy. Children with hereditary mevalonate kinase deficiency frequently exhibit febrile or afebrile seizures, underscoring the *in vivo* relevance of this metabolic pathway (34). Clinical studies on patients with drug-resistant epilepsy have demonstrated a significant negative correlation between serum CoQ10 levels and seizure frequency. Notably, the administration of either mevalonate or coenzyme Q10 has been shown to mitigate simvastatin-induced exacerbation of epileptic seizures and associated neuronal damage (58).

In this article, we review disorders of iron metabolism may underlie the mechanisms linking ferroptosis and seizures. However, how would ferroptosis function as a repeated seizure event of abnormal nervous system stress? Or, how might ferroptosis affect epilepsy progression?

### 3.2 The exacerbation of disease deterioration and adverse outcomes is facilitated by the progression of ferroptosis induced by epilepsy

It is crucial to clarify that iron overload does not solely manifest prior to the onset of epilepsy, and seizures often exacerbate the disruption of iron metabolism. Research has demonstrated that the ferroptosis inhibitor Fer-1 exhibits potential in ameliorating epileptiform seizures in rats with PTE, while also significantly safeguarding against seizure-induced cognitive impairment. These findings provide support for the pivotal role of ferroptosis in epilepsy pathology (59).

Firstly, seizure-induced chronic neuronal iron uptake may contribute to neuronal loss in temporal lobe epilepsy and hippocampal sclerosis (TLE-HS). Several studies have reported that seizures can activate the hif-1a/HO-1 pathway, leading to abnormal iron metabolism and increased Fe^2+^ accumulation in hippocampal neurons. Excessive Fe^2+^ accumulation can induce oxidative stress and cell damage, thereby triggering ferroptosis of hippocampal neurons and further promoting seizures (60). Secondly, sensitivity weighted imaging was employed to investigate alterations in whole-brain iron levels in patients with mesial temporal lobe epilepsy (MTLE) from central China. The findings revealed a redistribution of iron between subcortical and cortical structures, influenced by the progression of seizures (61). Microinjection of Fe^3+^ into the brain of an animal model for epilepsy resulted in glutamate release, which is known to be a systemic Xc^−^ inhibitor (62). However, it is well-established that elevated extracellular glutamate levels during seizures contribute to relapse and facilitate seizure onset as well as status epilepticus (SE) (63).

Furthermore, seizures induce an excessive production of ROS and promote ferroptosis, thereby exacerbating oxidative stress. This oxidative stress is particularly pronounced in brain tissues during epilepsy due to their high oxygen consumption, leading to increased generation of free radicals compared to other tissues and consequently aggravating neuronal cell death in various brain regions. It is closely associated with the development of comorbidities in epilepsy (64). For instance, researchers have observed activation of the ferroptosis process in a mouse model of Epilepsy-associated cognitive disorder (ECD), and inhibition of ferroptosis through pharmacological intervention or genetic manipulation targeting Lox can ameliorate ECD-related damage.

The alarming aspect is that Ferroptosis, which is believed to be exacerbated by seizures, is associated with sudden unexpected death in epilepsy (SUDEP) (65). Seizures are accompanied by elevated levels of intracellular free Fe^2+^ ions and deposition of hemosiderin. Existing reports suggest significant iron buildup in the brain and heart that is connected to epilepsy (66). Generalized tonic–clonic seizures (GTCS) present a considerable risk for SUDEP, affecting not only the brain but also causing cardiogenic dysfunction characterized by excessive iron accumulation and cardiomyopathy (IOC), along with electrical and mechanical abnormalities referred to as ‘epileptic heart,’ which carries a high likelihood of malignant bradycardia (67). Researchers induced SE in rats using pilocarpine and observed substantial accumulation of hemosiderin within cardiomyocytes, which was also correlated with an increase in spontaneous mortality (66). Therefore, hypoxia-ischemia–reperfusion secondary to status epilepticus can not only induce up-regulation of P-glycoprotein (P-gp) and down-regulation of Kir channels, but also impact cardiac repolarization associated with epileptic heart and iron accumulation as well as ptosis associated with ischemic optic neuropathy (ION). These two mechanisms, triggered by the same convulsive stress, can simultaneously lead to severe cardiac dysfunction. Furthermore, ferroptosis is a common consequence of both brain and cardiac hypoxia. This dual effect induces neurodegeneration and epileptogenesis in heart failure (epileptic heart) while increasing the high risk of SUDEP (68, 69).

**Figure 4:**
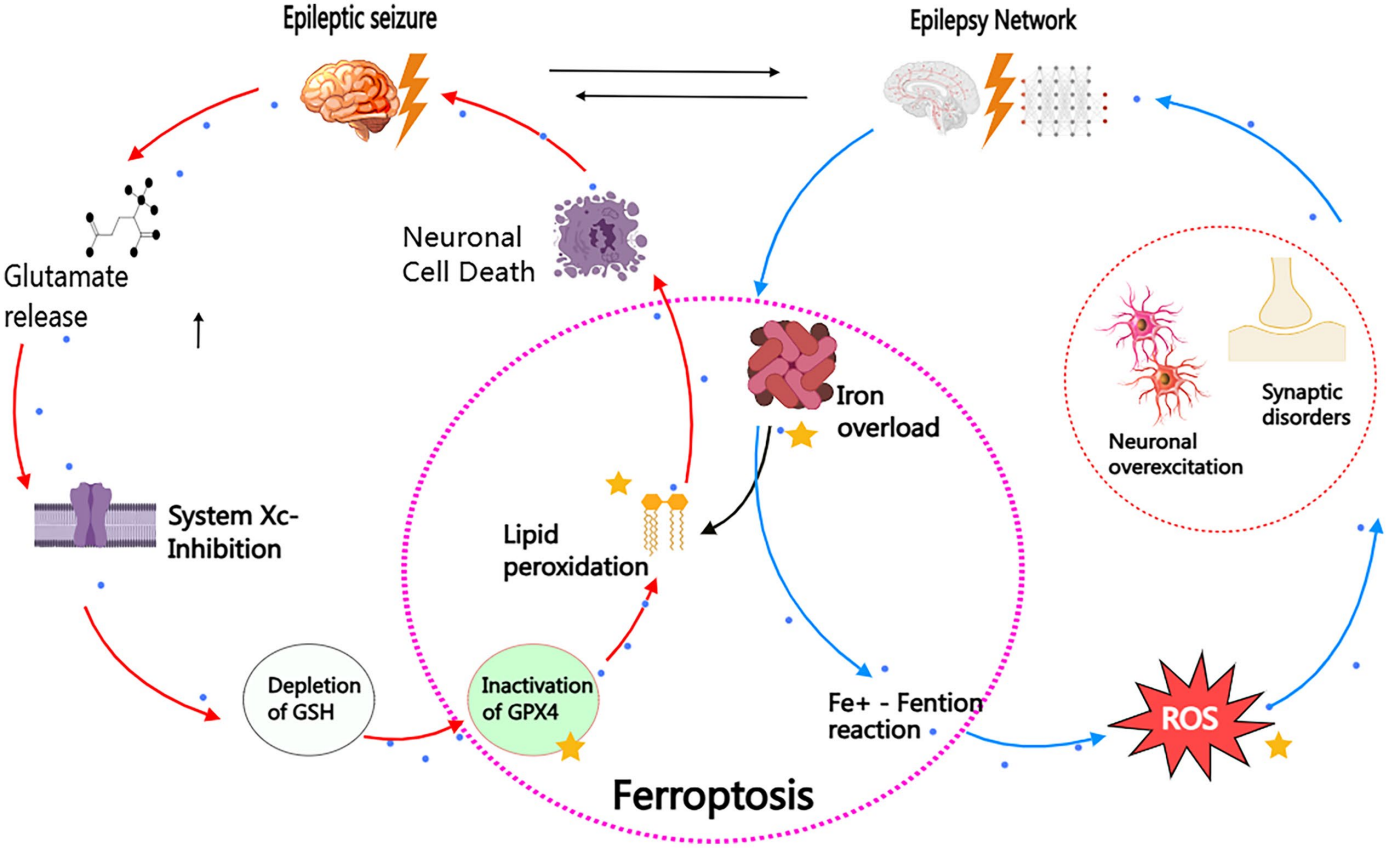
Bidirectional pathogenic loop between ferroptosis and epilepsy. This schematic illustrates the self-reinforcing cycle linking ferroptosis and epilepsy through two interconnected axes: I. Epilepsy. Ferroptosis Pathway (Red Arrows) Seizure activity triggers glutamate excitotoxicity, inhibiting the cystine/glutamate antiporter (System Xc-). This depletes glutathione (GSH), inactivating GPX4 and enabling iron-dependent lipid peroxidation. Subsequent membrane rupture and neuronal death further potentiate epileptogenesis. II. Ferroptosis Epilepsy Pathway (Blue Arrows) Iron overload (e.g., post-hemorrhagic or metabolic) generates hydroxyl radicals (OH) via Fenton reactions. Reactive oxygen species (ROS) hyperexcite neurons through Nav channel dysregulation and mitochondrial dysfunction, establishing hyper-synchronous networks that lower seizure thresholds. Therapeutic Intervention Points (Yellow Stars): (1) Iron chelators (e.g., DFO): Block Fe^2−^-mediated ROS production. (2) GPX4 activators (e.g., Selenium): Restore lipid peroxide detoxification. (3) Radical-trapping antioxidants (e.g. Fer-1): Terminate lipid peroxidation chain reactions. The vicious cycle highlights ferroptosis as both cause and consequence of epilepsy, providing mechanistic targets for clinical intervention.

As mentioned earlier, our discussion highlights an important point: it is not advisable to overemphasize the direct causal relationship between epilepsy and ferroptosis. The reason is that ferroptosis may act as an auxiliary factor in the pathogenesis of epilepsy, potentially promoting the occurrence of epilepsy; at the same time, the repeated attacks of epilepsy may exacerbate the ferroptosis process, thus forming a vicious cycle of mutual causation, which may further worsen the clinical outcomes of the disease. Figure 4 illustrates the bidirectional pathological interaction between ferroptosis and epilepsy. This discovery actually reveals the huge potential and feasibility of ferroptosis process in epilepsy as a diagnostic biomarker and therapeutic target.

### 3.3 Clinical evidence of ferroptosis in human epilepsy

Emerging clinical studies have begun to reveal potential ferroptosis signatures in patients with epilepsy, though evidence remains preliminary. For instance, SWI has identified abnormal iron accumulation in the hippocampus and cortex of patients with MTLE, correlating with seizure frequency and disease duration (70, 71). Victoria Martella et al. further demonstrated visual biomarkers of iron deposition within the pulvinar region of the thalamus in epilepsy patients using SWI (72). Metabolomic analyses of cerebrospinal fluid (CSF) from drug-resistant epilepsy patients show alterations in oxidative stress markers (e.g., lipid peroxidation products), suggesting possible ferroptotic activity (73, 74). Additionally, hematological studies in pediatric epilepsy patients report GSH depletion and GPX4 inactivation (75).

However, critical limitations must be acknowledged: ① Spatiotemporal Gaps: Current imaging detects iron accumulation but cannot confirm dynamic ferroptosis progression. The causal relationship between iron deposition and epileptogenesis requires longitudinal studies. ② Biomarker Specificity: Altered oxidative markers in CSF/blood may reflect general neurodegeneration rather than ferroptosis specifically. Validation with direct ferroptosis indicators (e.g., ACSL4, PUFA-OOH) is needed. ③ Therapeutic Translation: No clinical trials have yet tested ferroptosis inhibitors in epilepsy patients, leaving human efficacy unproven despite promising preclinical data.

Despite these challenges, existing evidence provides crucial foundations. Iron deposition patterns observed on SWI may serve as non-invasive prognostic tools for epilepsy progression. Additionally, deficits in GSH and GPX4 highlight testable hypotheses for the personalization of antioxidant therapy. Furthermore, multi-omics approaches that integrate iron-metabolomics, lipidomics, and epigenomics could uncover definitive ferroptosis signatures. Future studies should prioritize brain tissue biopsies, obtained from epilepsy surgery, to map the molecular drivers of ferroptosis and launch phase I/II trials of repurposed iron chelators, such as deferiprone, in drug-resistant cohorts.

## 4 Potential role of ferroptosis mechanism in the treatment of epilepsy

### 4.1 Application of ferroptosis inhibitors and associated agents in the treatment of epilepsy

Firstly, the exploration of the application of ferroptosis inhibitors in the treatment of epilepsy can be traced back to some fundamental research. Deferoxamine (DFO), which is an iron chelator capable of effectively scavenging iron, has been found that its treatment of epilepsy induced by ferric chloride can reduce local transferrin and significantly inhibit epilepsy (76). Secondly, considering the inseparable relationship between ferroptosis and ROS, the use of antioxidants (e.g., vitamin E, melatonin, etc.) (77–79), ferroptosis inhibitors such as ferrostain-1 (Fer − 1), and/or iron chelators can prevent brain iron accumulation and the neuroprotective role of ROS, thereby blocking the pathological process of epilepsy to a certain extent (80).

For instance, in a mouse model of PTZ-induced seizures and pilocarpine-induced seizures, the administration of Fer-1 significantly mitigated seizure severity and frequency while reducing iron accumulation in the hippocampus (81). In another experimental model of post-traumatic epilepsy induced by stereotactic injection of 50 mM FeCl_3_ into the somatosensory cortical area, Fer-1 exhibited a notable protective effect against acute seizures and memory decline (59). Moreover, in a previously described rat model of pilocarpine-induced TLE, Klotho overexpression was induced in the hippocampus using an adeno-associated viral vector delivery system. This approach not only inhibited ferroptosis and iron overload but also regulated the expression of divalent metal transporter 1 and ferrotransporter, both associated with iron accumulation in the hippocampus. Consequently, it effectively ameliorated cognitive deficits and demonstrated neuroprotective effects. Importantly, Klotho significantly elevated GPX4 and GSH levels while suppressing ROS levels. In summary, this protein alleviates cognitive deficits and exerts neuroprotective effects by inhibiting ferroptosis in a rat model of TLE (82, 83). Coenzyme Q10 (CQ 10) is a compound renowned for its anti-inflammatory and antioxidant properties. Studies have demonstrated that appropriate CQ 10 supplementation also exhibits an inhibitory effect on ferroptosis (84). Furthermore, a separate study confirmed the involvement of ferroptosis in the pathogenesis of epilepsy associated with mitochondrial disease and revealed that alpha-tocotrienol quinone (EPI-743) effectively ameliorated ferroptosis in cells derived from patients with five distinct childhood epilepsy syndromes in a dose-dependent manner by reducing lipid peroxidation (LPO) and 15-hydroxyditetraenoic acid (15-HETE) levels (17).

Furthermore, it is crucial to consider the interaction between ferroptosis inhibitors and traditional antiepileptic drugs. The mechanism of action of traditional antiepileptic drugs is intricate, encompassing enhancement of GABA-mediated inhibitory neurotransmission, inhibition of excitatory amino acid release or function, blockade of voltage-dependent sodium channels, and regulation of calcium channels. However, emerging evidence suggests that apart from these conventional mechanisms, iron metabolism imbalance and ferroptosis may significantly impact the efficacy of antiepileptic drugs. For instance, certain antiepileptic drugs like sodium valproate (VPA) exhibit notable antioxidant activity which effectively reduces free radical generation and consequently inhibits ferroptosis. In a mouse model with kainic acid-induced epilepsy, VPA not only markedly decreased lipid peroxide levels but also suppressed Ptgs2 mRNA expression. Importantly, the positive correlation observed between VPA’s inhibitory effect on ferroptosis and its antiepileptic effect strongly indicates that modulation of ferroptosis might be an important avenue for harnessing the therapeutic potential of antiepileptic drugs (85). Conversely, the occurrence of ferroptosis could impair the effectiveness of certain antiepileptic drugs as well. Specifically, excessive iron accumulation and subsequent induction of ferroptosis can trigger lipid peroxidation in neuronal membranes leading to alterations in their physical and chemical properties. Such changes undoubtedly influence drug penetration and targeting within cells thereby posing a potential threat to therapeutic efficacy (86).

Furthermore, numerous studies have revealed that specific antiepileptic drugs may exert their unique pharmacological effects by directly or indirectly interfering with the signaling pathway of ferroptosis. For instance, topiramate, a widely recognized broad-spectrum antiepileptic drug, not only possesses significant antioxidant and anti-inflammatory properties but also effectively mitigates cellular oxidative stress, which is one of the key triggers of ferroptosis (87). Additionally, lamotrigine not only inhibits excitatory synaptic transmission but also fine-tunes intracellular iron ion concentration to potentially reduce the occurrence of ferroptosis events (88). Table 1 provides a comprehensive summary of drugs that have the potential to enhance epilepsy treatment through their impact on ferroptosis.

**Table 1:** Antiferroptotic mechanisms may exert an antiepileptic effect.

Drugs	Mechanisms of intervention	Categories	References
Valproic acid (VPA)	VPA contributes to iron metabolism in epilepsy, leading to the generation of non-transferrin-bound iron (NTBI) and an elevation in oxidative stress levels. Additionally, there was an observed upregulation of SLC7A11 protein expression within hippocampal neurons.	Antiepileptic drug/System Xc^−^ modulator (With antioxidant and ferroptosis-regulating properties)	(87)
Topiramate	Topiramate has antioxidant and anti-inflammatory properties and can reduce the effects of oxidative stress in cells	Broad-spectrum antiepileptic drug (Free radical scavenger and inflammation modulator)	(90)
Anticonvulsant	Antioxidant effects of lamotrigine on pilocarpine-induced status epilepticus in mice	Sodium channel-blocking antiepileptic drug (Oxidative stress mitigator)	(91)
Selenium	Reinforced upregulation of the antioxidant GPX4	Essential trace element supplement (GPX4 activator)	(33)
Ferrostatin-1 (Iron-1)	Ferstatin-1 effectively mitigates hippocampal hypertrophy in KA-treated rats by downregulating GPX4 expression, restoring GSH levels, and attenuating lipid peroxidation and iron accumulation. Furthermore, in TLE rats, iron-statin-1 prevented KA-induced hippocampal neuronal loss and restored cognitive function	Ferroptosis-specific inhibitor (Radical-trapping antioxidant)	(62, 84)
Edaravone	It plays an anti- ferroptosis role by scavenging lipid peroxides	Clinically approved antioxidant (Lipid peroxide scavenger)	(81)
Deferoxamine (DFO)	It can reduce the iron deposition in the mitochondria of cerebral cortical neurons in epileptic rats	Iron chelator (High-affinity ferric iron binder)	(79)
Dehydroepiandrosterone (DHEA)	The administration of DHEA has been demonstrated to exert inhibitory effects on lipid peroxidation, protein oxidation, and Na+/K + -ATPase activity in individuals with epilepsy. Furthermore, DHEA demonstrates the ability to alleviate oxidative stress and cellular damage associated with iron-induced epilepsy through the activation of the NRF2/ARE signaling pathway leading to apoptosis.	Neurosteroid hormone (Multi-pathway antioxidant and anti-apoptotic agent)	(55)
Vitamins E and C, melatonin, vanillin	Exhibiting antioxidant properties, effectively scavenging free radicals and mitigating the production of ROS	Dietary antioxidant supplements (Chain-breaking radical scavengers)	(80–82)
α-phenyltert-butylnitroone (PBN)	Reduce the oxidative damage of epileptic nerve cells induced by iron	Nitrone-based radical trapping agent (Reactive nitrogen species scavenger)	(83)
EPC-K1	Hydroxyl radical scavengers protect nerve cell membrane from oxidation and prevent epileptic discharge caused by iron ions	Vitamin-derived compound (Membrane-targeted antioxidant)	(68)
Adenosine chloride (Cl-Ado) or adenosine (Ado)	The administration of Cl-Ado or Ado effectively attenuated or delayed the initiation of FeCl3-induced epileptic discharges through scavenging hydroxyl radicals, thereby exhibiting its anticonvulsant properties.	Endogenous purine nucleoside (Anticonvulsant and radical scavenger)	(69)
Fisetin	Non-oxetine exhibits the potential to attenuate lipid peroxidation and preserve the activities of Na + and K + -ATpase in patients suffering from post-traumatic epilepsy. Simultaneously, fisetin demonstrates its anti-epileptic efficacy in an iron-induced epilepsy rat model by inhibiting oxidative stress.	Flavonoid polyphenol (Dual inhibitor of ferroptosis and oxidative stress)	(52)
Coenzyme Q10 (CoQ10)	Studies have demonstrated that appropriate supplementation of coenzyme Q10 also exhibits an inhibitory effect on ferroptosis. Cases of epilepsy caused by CoQ10 deficiency have been reported	Mitochondrial electron transporter (Lipid-soluble antioxidant and membrane stabilizer)	(87)

In conclusion, there exists an intricate and interconnected network between ferroptosis and antiepileptic drugs. A comprehensive exploration into the role of the ferroptosis mechanism within this network in epilepsy treatment will undoubtedly unveil novel strategies and targets while promoting more efficient and precise therapeutic approaches. Therefore, future research should focus on elucidating the specific molecular mechanisms underlying the interaction between ferroptosis and antiepileptic drugs to provide a robust scientific foundation and practical guidance for enhancing treatment efficacy and improving quality of life among epilepsy patients.

### 4.2 Implications of AMD research for ferroptosis-targeted epilepsy treatment

**Figure 5:**
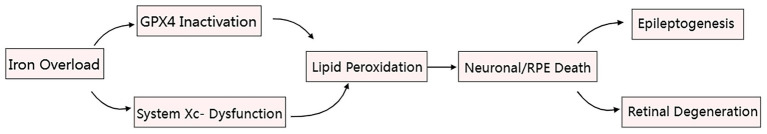
Iron-mediated ferroptosis core pathways in epilepsy (hippocampal neurons) and AMD (retinal pigment epithelium). Therapeutic targets (red stars): ① Iron chelation, ② GPX4 activation, ③ Antioxidant delivery.

When exploring the potential role of ferroptosis mechanisms in epilepsy treatment, valuable insights can be gained from the study of ferroptosis in other neurodegenerative diseases. Age-related macular degeneration (AMD), a neurodegenerative condition, is closely associated with ferroptosis and provides important references for epilepsy treatment (89) (Shared ferroptotic pathways are illustrated in Figure 5). The pathological features of AMD, including iron accumulation in the retina, lipid peroxidation, and decreased GPX4 activity, are highly consistent with the mechanisms of ferroptosis (89). Interventions used in AMD research, such as iron chelators, ferroptosis inhibitors, and activation of the Nrf2 pathway, offer potential strategies for epilepsy treatmen.

Moreover, the experience gained from AMD research also provides important references for developing epilepsy treatments targeting ferroptosis. In terms of diagnosis, a hippocampal iron quantification MRI standard could be developed, for example, setting an iron concentration threshold of >1.2 mg/g to identify high-risk epilepsy patients. Regarding drug delivery strategies, a cranial sustained-release system loaded with ferroptosis inhibitors (such as Ferrostatin-1) could be developed, mimicking the intravitreal injections used in AMD, to achieve localized drug release and enhance therapeutic efficacy (90). In clinical trial design, a composite endpoint approach used in AMD research could be adopted to evaluate both seizure frequency (primary endpoint) and changes in SWI iron signals (secondary endpoint), providing more comprehensive evidence for the study of ferroptosis mechanisms. Although these measures have not yet been validated in epilepsy, these translational concepts have the potential to offer new ideas and methods for epilepsy treatment targeting ferroptosis, from diagnostic assessment to therapeutic strategies, and to promote the clinical application of relevant treatment approaches.

## 5 Research status and challenges

Although there has been some progress in exploring the role of ferroptosis in the pathogenesis of epilepsy in recent years, the current research still faces many limitations, which greatly limit our depth and breadth of our comprehensive cognition and practice application in this area. The first problem is that a large number of studies focus on external and animal models, and the direct clinical evidence of epilepsy patients is inadequate. It is true that the experiment and animal model of vitro provides us with valuable mechanism exploration and drug screening platform, but these results often face many uncertainties in the transformation of the complex human physiological environment. Especially in the context of epilepsy, a multi-factor disease, the exploration of a single mechanism is difficult to fully reveal its intricate pathological process. Second, existing research tends to focus on a specific link or signaling pathway of iron death, and the interaction between it and its disease mechanism is not enough. For example, iron death, inflammatory response, oxidative stress and mitochondrial dysfunction may be synergistic in the onset of epilepsy, but the correlation and impact of these factors have not been systematically studied. The fragmentation of this study undoubtedly prevents our understanding of the comprehensive and systematic understanding of iron deaths in the mechanism of epilepsy.

In addition, the complexity of the protein and signaling pathways of iron deaths increased the difficulty of the study. Currently, the study focuses on several major proteins and molecular markers, such as GPX4, Ferritin and ROS, but do not explore other potential key molecules. Because iron death is a process of multi-step, multi-signal channel participation, the neglect of any link can lead to misunderstanding of the overall mechanism. Moreover, the study of iron death in epilepsy treatment is still in the preliminary exploration phase, and there is a lack of systematic clinical trial data. Although some iron death inhibitors show certain therapeutic potential in animal models, the applicability, side effects and long-term effects of different types of epilepsy are not fully verified. Moreover, most studies have focused on a particular drug or treatment strategy, but failed to provide multiple strategies for comparison and combined application research data.

### 5.1 Prospects for future research directions

Although ferroptosis research has demonstrated promising prospects in the field of epilepsy, scientists still need to overcome current limitations and adopt diversified and interdisciplinary comprehensive research methods. These include integrating *in vitro* and *in vivo* experiments, conducting extensive clinical research, and fostering collaboration among multidisciplinary teams. By doing so, we can gradually unravel the intricate mechanisms underlying ferroptosis in the pathogenesis of epilepsy, thereby establishing a more robust theoretical foundation for its diagnosis and treatment. Future investigations should prioritize several core directions:

1. To investigate the distinct role of ferroptosis in various types of epilepsy: Given the substantial heterogeneity observed in epilepsy, different types may exhibit diverse pathogenic mechanisms (91, 92). Therefore, it is imperative to comprehensively explore whether discrepancies exist in the involvement of ferroptosis across different types of epilepsy, aiming to unveil the universal principles governing ferroptosis action and its specific efficacy for particular epileptic conditions.
2. Investigating the combined effect of ferroptosis inhibitors and existing antiepileptic drugs aims to explore the potential benefits of comprehensive treatment in terms of improving drug efficacy and reducing side effects. This research not only contributes to optimizing clinical treatment plans but also sheds light on the underlying role of ferroptosis in epilepsy therapy.
3. Discovery and application of biomarkers: Future studies should aim to identify biomarkers associated with ferroptosis for early diagnosis, disease progression monitoring, and evaluation of treatment efficacy in epilepsy (93). This will provide valuable insights for personalized treatment strategies and may also unveil novel therapeutic targets.
4. Clinical trials and translational research: Despite significant progress in animal models regarding ferroptosis-related studies, further validation is required before clinical application can be realized. Large-scale clinical trials are essential to generate robust evidence that can determine the actual effectiveness and safety profile of ferroptosis inhibitors in patients with epilepsy.

Through comprehensive exploration and meticulous research in the aforementioned fields, we aim to unveil the fundamental mechanisms underlying ferroptosis throughout the entire trajectory of epilepsy onset, progression, and treatment. Moreover, our endeavors will pave the way for unprecedented therapeutic perspectives for individuals with epilepsy while fostering the development of more efficient and targeted treatment protocols.

## Glossary

ROS: Reactive Oxygen Species
Tf: Transferrin
TfR1: Transferrin Receptor 1
BMECs: Brain Microvascular Endothelial Cells
DCYTB: Duodenal Cytochrome B
STEAP3: Six-Transmembrane Epithelial Antigen of Prostate 3
DMT1: Divalent Metal Transporter 1
LIP: Labile Iron Pool
FPN: Ferroportin
GSH: Glutathione
GPX4: Glutathione Peroxidase 4
PUFAs: Polyunsaturated Fatty Acids
NADPH: Nicotinamide Adenine Dinucleotide Phosphate
GSSG: Oxidized Glutathione
RSL3: RAS-Selective Lethal compound 3
PTE: Post-Traumatic Epilepsy
GABA: Gamma-Aminobutyric Acid
TSC: Tuberous Sclerosis Complex
FCD: Focal Cortical Dysplasia
TLE: Temporal Lobe Epilepsy
HS: Hippocampal Sclerosis
LPCAT3: Lysophosphatidylcholine Acyltransferase 3
MTLE: Mesial Temporal Lobe Epilepsy
SE: Status Epilepticus
SUDEP: Sudden Unexpected Death in Epilepsy
GTCS: Generalized Tonic-Clonic Seizures
IOC: Iron Overload Cardiomyopathy
P-gp: P-glycoprotein
ION: Ischemic Optic Neuropathy
DFO: Deferoxamine
VPA: Valproic Acid
NTBI: Non-Transferrin Bound Iron
Fer-1: Ferrostatin-1
CQ10: Coenzyme Q10
LPO: Lipid Peroxidation
15-HETE: 15-Hydroxyeicosatetraenoic Acid
AEDs: Antiepileptic Drugs
SWI: Susceptibility Weighted Imaging
AMD: Age-related Macular Degeneration
RPE: Retinal Pigment Epithelium
OCT: Optical Coherence Tomography
CoQ10: Coenzyme Q10
ACSL4: Acyl-CoA Synthetase Long Chain Family Member 4
MVA: mevalonate
